# Dimensionality of luminescent coordination polymers of magnesium ions and 1,1′-ethynebenzene-3,3′,5,5′-tetracarboxylic acid modulated by structural inducing agents†

**DOI:** 10.1039/c8ra04875d

**Published:** 2018-07-16

**Authors:** Zhu-Xi Yang, Yin Qian, Jing-Wei Yu, Lu Zhai, Wen-Wei Zhang, Xiao-Ming Ren

**Affiliations:** State Key Laboratory of Materials-Oriented Chemical Engineering, College of Chemistry & Molecular Engineering, Nanjing Tech University Nanjing 210009 P. R. China zhailu@njtech.edu.cn xmren@njtech.edu.cn; State Key Laboratory of Coordination Chemistry, School of Chemistry and Chemical Engineering, Nanjing University Nanjing 210023 P. R. China; College of Materials Science & Engineering, Nanjing Tech University Nanjing 210009 P. R. China

## Abstract

Solvothermal reactions of aromatic 1,1′-ethynebenzene-3,3′,5,5′-tetracarboxylic acid (H_4_EBTC) and Mg^2+^ salts in the presence of different supporting ligands afforded the coordination polymers [Mg(H_2_EBTC)(DMF)_2_(H_2_O)_2_] (1), [Mg_3_(HEBTC)_2_(H_2_O)_4_]·solvent (2) and [Mg_2_(EBTC)(H_2_O)_5_]·solvent (3). The crystal structures of 1–3 were determined by the single crystal X-ray diffraction technique, where CP 1 showed a one-dimensional zigzag MgO_6_ coordination octahedral chain structure; 2 exhibited a two-dimensional MgO_6_ coordination octahedral framework with trinuclear [Mg_3_(COO)_6_] SBUs, and 3 featured a three-dimensional MgO_6_ coordination octahedral framework with binuclear [Mg_2_O(COO)_2_] SBUs. The various structures in CPs 1–3 of Mg^2+^ ions with the H_4_EBTC ligand were ascribed to the conformational flexibility and the coordination mode diversity of the H_4_EBTC ligand. Interestingly, the zwitterionic supporting ligand 2-aminoterephthalic acid or 4-aminobenzenesulphonic acid played a vital role in the initial formation process of nuclear crystals but only as a structural induction agent, which modulated the dimensionality of these Mg^2+^-based CPs. Additionally, the three CPs emitted bright blue luminescence at ambient conditions, and the emission lifetimes and absolute quantum yields were also investigated.

## Abstract 2

Crystal structure diversity and dimensionality of three Mg^2+^-based CPs, which emit bright ligand-based luminescence at ambient conditions, are modulated by reaction temperature and structural induction agents.

## Graphical abstract

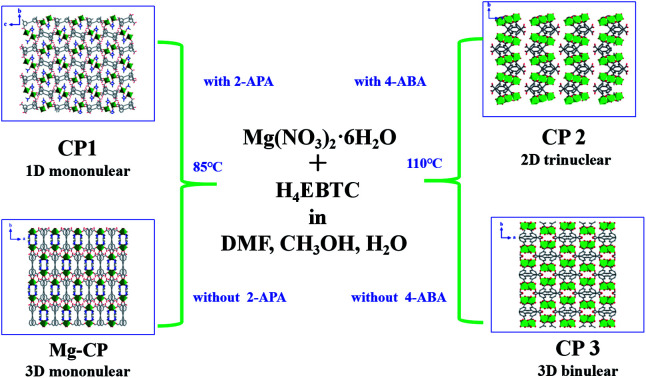

## Introduction

As an emerging class of crystalline solids with intrinsically well-organized host structures, functional coordination polymers (CPs) have attracted extensive research interest in the areas of inorganic chemistry, coordination chemistry, crystal engineering and materials science.^1–5^ In this context, the structural design and controllable synthesis of functional CPs have become some of the main themes of researchers in recent years, and researchers have explored various synthetic strategies to achieve target CPs with the desired structure and functionality. As is well-known, the structural diversity of CPs is dependent on many factors including the molecular structure of the organic ligand (molecular configuration and conformation), nature of the central metal ion (electron configuration and ionic radius, which predominate the coordination geometry and coordination number of a metal ion), ratio of metal ion to ligand, pH of the reaction solution, anion type of the metal salt in the starting materials and supporting ligand or template as well as the reaction temperature and time.^6–15^ In most cases, a subtle change in one of the factors mentioned above can lead to a drastic change in the dimensionality and topology of the final crystalline product; unfortunately, it is unclear how the subtle change in a certain factor in a solvothermal process plays a role in the dimensionality and topology of the final product. Particularly, the effect of a supporting ligand or template on the structural transformation still remains largely unexplored to date. For the crystal engineering of CPs, undoubtedly, it is rather important to better understand the mechanism by which a certain factor affects the crystal structure of the product.

It is well-known that an aromatic multicarboxylic acid ligand possesses several COOH groups in a molecule and in general, these COOH groups not only adopt abundant coordination modes, but also show a tunable degree of deprotonation. Such a unique structure and binding features of an aromatic multicarboxylic acid ligand usually give rise to crystal structure diversity of the final CP product.^16–18^ It is also worth noting that the introduction of a supporting ligand in the solvothermal reaction system can result in a dramatic change in the crystal structure of the final CP product; the supporting ligand acts as the auxiliary ligand in some cases, but it only serves as a template (or a structural induction agent, abbr. as SIA) in other cases.^19,20^ In the second situation, the role played by SIA in the initial formation and growth of nuclear crystals is unclear, and this deserves further study.

In the field of CPs, alkaline earth-metal-based CPs represent an important subcategory of CPs with interesting structures and fascinating physical properties.^21^ With respect to the transition/rare earth metal-based CPs, the alkaline earth–metal-based CPs have a series of advantages such as low/no toxicity and low cost due to their abundance in earth;^22^ however, they have received less attention.^23^ As a cheap and abundant ion source, the alkaline earth metal ions with a closed-shell electronic configuration are suitable for the preparation of luminescent CPs that have the characteristics of ligand emission.^24^ In this regard, we have been exploring the synthesis approach and photoluminescence properties of CPs of Mg^2+^ ion with the π-electron-rich alkyne-functionalized tetracarboxylic acid ligand 1,1′-ethynebenzene-3,3′,5,5′-tetracarboxylic acid (H_4_EBTC);^25^ previous studies have demonstrated that when the H_4_EBTC ligand is partly or fully deprotonated, it can act as an excellent phosphor, and it simultaneously emits ligand-based fluorescence and phosphorescence within a colorless Mg^2+^-based CP at room temperature.^25^

**Scheme 1:**
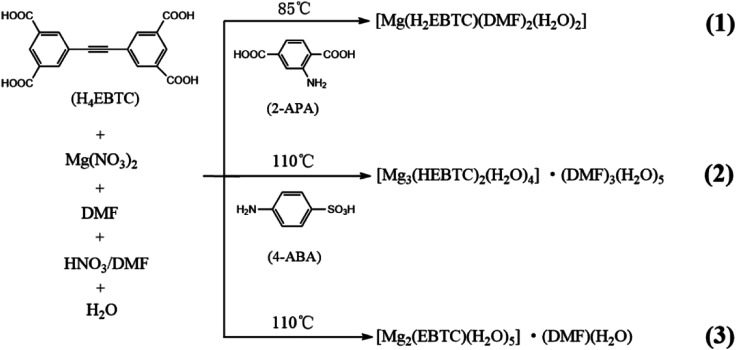
Syntheses of CPs 1–3.

We aim at further exploring and better understanding the self-assembly behaviors of Mg^2+^ ions with an excellent luminescence ligand H_4_EBTC in the presence of a supporting ligand under solvothermal conditions. Fortunately, by adjusting the reaction temperatures and with or without the help of a supporting ligand, we have successfully obtained three CPs of Mg^2+^ ion with the H_4_EBTC ligand having different degrees of deprotonation, and the CPs are [Mg(H_2_EBTC)(DMF)_2_(H_2_O)_2_] (1), [Mg_3_(HEBTC)_2_(H_2_O)_4_]·(DMF)_3_(H_2_O)_5_ (2) and [Mg_2_(EBTC)(H_2_O)_5_]·(DMF)(H_2_O) (3) (Scheme 1). It is found that SIA plays an important role in modulating the dimensionality of CPs, and the partly/fully deprotonated H_4_EBTC ligand shows diverse coordination modes with Mg^2+^ ions and emits bright blue fluorescence in the three CPs at an ambient temperature.

## Experimental section

### Reagents and materials

All reagents and materials are of analytical grade and used as received from commercial sources without further purification. H_4_EBTC was synthesized according to the method published.^26^

### Chemical analysis and physical measurements

IR spectra were obtained on a NICOLET iS10 spectrometer in the 4000–400 cm^−1^ region. Powder X-ray diffraction (PXRD) data were recorded on a Bruker D8 Discover diffractometer with Cu Kα (*λ* = 1.54056 Å) radiation and a scan speed of 5° min^−1^ and a step size of 0.02° in 2*θ*. Elemental analyses of C, H, and N were determined using a PerkinElmer 2400C automatic analyzer. The luminescent images were obtained using a Nikon polarizing optical microscope (NIS-Elements Documentation) equipped with an ultraviolet light source (*λ*_ex_ = 330–380 nm). Thermal gravimetric analyses (TGA) were performed using a DTA-TGA 2960 thermogravimetric analyzer in a nitrogen atmosphere with a heating rate of 10 °C min^−1^ from 25 to 800 °C.

Steady state emission and excitation spectra were recorded for the solid samples on an F-7000 FL spectrofluorometer equipped with a 150 W Xenon lamp as an excitation source at room temperature. The photomultiplier tube (PMT) voltage was 700 V for all measurements. The scan speed was 1200 nm min^−1^. The room temperature luminescence study was performed on a Fluorolog-3-TAU fluorescence spectrophotometer. The fluorescence lifetime measurement was obtained by a single-photon counting spectrometer using an Edinburgh FLP920 spectrometer equipped with a continuous Xe900 xenon lamp. Absolute photoluminescence quantum yields were measured on a Steady-State and Time-Resolved Fluorescence Spectrofluorometer.

### Preparation of 1–3

#### [Mg(H_2_EBTC)(DMF)_2_(H_2_O)_2_] (1)

A mixed solution containing Mg(NO_3_)_2_·6H_2_O (8 mg, 0.056 mmol), H_4_EBTC (5 mg, 0.014 mmol), 2-aminoterephthalic acid (2-APA) (3 mg, 0.017 mmol), DMF (0.4 mL), CH_3_OH (0.10 mL), HNO_3_ (0.04 mL, 1 M in DMF) and H_2_O (0.10 mL) was sealed in a 10 mL Teflon-lined autoclave and heated to 85 °C for 24 h. Colorless block-shaped crystals were achieved after the Teflon-lined autoclave slowly cooled to an ambient temperature (yield: *ca.* 58% based on H_4_EBTC). Anal. elemental analysis: calcd for C_24_H_26_MgN_2_O_12_: C, 51.58; H, 4.69; N, 5.01. Found: C, 51.62; H, 4.98; N, 5.56. Selected IR data (KBr pellet, cm^−1^): 3360 (b), 1567 (m), 1432 (m), 1378 (s), 1101 (m), 775 (w), 704 (w).

#### [Mg_3_(HEBTC)_2_(H_2_O)_4_]·(DMF)_3_(H_2_O)_5_ (2)

A mixed solution with Mg(NO_3_)_2_·6H_2_O (8 mg, 0.056 mmol), H_4_EBTC (5 mg, 0.014 mmol), 4-aminobenzenesulphonic acid (4-ABA) (3 mg, 0.017 mmol), DMF (0.4 mL), CH_3_OH (0.10 mL), HNO_3_ (0.04 mL, 1 M in DMF) and H_2_O (0.10 mL) was sealed in a 10 mL Teflon-lined autoclave and heated to 110 °C for 24 h. Colorless block-shaped crystals were achieved after the Teflon-lined autoclave slowly cooled to room temperature (yield: *ca.* 60% based on H_4_EBTC). Anal. elemental analysis: calcd for C_36_H_22_Mg_3_O_20_: C, 46.72; H, 4.62; N, 3.63. Found: C, 46.35; H, 4.66; N, 3.23. Selected IR data (KBr pellet, cm^−1^): 3207 (b), 1557 (m), 1428 (m), 1366 (s), 1102 (m), 777 (w), 711 (w).

#### [Mg_2_(EBTC)(H_2_O)_5_]·(DMF)(H_2_O) (3)

A mixed solution with Mg(NO_3_)_2_·6H_2_O (8 mg, 0.056 mmol), H_4_EBTC (5 mg, 0.014 mmol), DMF (0.4 mL), CH_3_OH (0.10 mL), HNO_3_ (0.04 mL, 1 M in DMF) and H_2_O (0.10 mL) was sealed in a 10 mL Teflon-lined autoclave and heated to 110 °C for 24 h. Colorless block-shaped crystals were achieved after the Teflon-lined autoclave slowly cooled to room temperature (yield: *ca.* 60% based on H_4_EBTC). Anal. Elemental analysis: calcd for C_18_H_16_Mg_2_O_13_: C, 43.48; H, 2.41; N, 4.34. Found: C, 43.20; H, 2.27; N, 4.33. Selected IR data (KBr pellet, cm^−1^): 3226 (b), 1567 (m), 1430 (m), 1361 (s), 1104 (m), 772 (w), 705 (w).

### Crystallographic analyses

Suitable single crystals of 1–3 were selected under an optical microscope and glued to thin glass fibers. Single crystal X-ray diffraction data were obtained on a Bruker Smart Apex II CCD diffractometer using graphite monochromated Mo/Kα radiation (*λ* = 0.71073 Å). Data reductions and absorption corrections were performed with the SAINT^27^ and SADABS2 (ref. 28) software packages, respectively. Structures were solved by a direct method using the SHELXL software package.^29^ The non-hydrogen atoms were anisotropically refined using the full-matrix least-squares method on *F*^2^. All hydrogen atoms were placed at the calculated positions and refined riding on the parent atoms. In the crystal structure of 1, one of the two crystallographically different DMF molecules showed disorder, and the corresponding atoms were refined using split-atom models; the occupied site factor was refined. In the crystal structures of 2 and 3, the frameworks contained severely disordered lattice solvents, and the large volume fractions of the disordered solvents in the lattices could not be modeled in terms of atomic sites and were treated using the SQUEEZE routine in the PLATON software package.^30^ The solvents were accounted for both 2 and 3 using SQUEEZE^31^ implemented in PLATON, which calculates the electron densities in the unit cells and accounts for them in the refinement. This electron density was assigned as some specific solvent content.

CCDC 1838042 (1), 1573136 (2) and 1573138 (3) contain the supplementary crystallographic data for this paper. The crystallographic data and details of structural refinements are listed in Table 1, and the selected bond distances and angles are listed in Table S1 in the ESI† for 1–3.

**Table 1:** Crystallographic data and structure refinement for 1–3

Compound	1	2	3
Formula	C_24_H_26_MgN_2_O_12_	C_36_H_22_Mg_3_O_20_·solvent	C_18_H_16_Mg_2_O_13_·solvent
Formula weight	558.78	847.46	488.93
CCDC no.	1838042	1573136	1573138
Temp. (K)	295(2)	173(2)	295(2)
Wavelength (Å)	0.71073	0.71073	0.71073
Crystal size/mm	0.21 × 0.18 × 0.16	0.15 × 0.15 × 0.12	0.15 × 0.15 × 0.12
Crystal system	Monoclinic	Monoclinic	Monoclinic
Space group	*P*2_1_/*n*	*P*2_1_/*c*	*C*2/*c*
*a*/Å	8.0079(7)	14.726(2)	16.8934(15)
*b*/Å	16.8300(15)	15.126(2)	14.1990(13)
*c*/Å	19.0430(16)	13.5397(18)	12.0673(11)
*α*/Å	90	90	90
*β*/Å	100.254(2)	116.871(6)	115.993(2)
*γ*/Å	90	90	90
*V*/Å^3^	2525.5(4)	2690.3(6)	2601.8(4)
*Z*	4	2	4
*F*(000)	1168	868	1008
*θ*_min,max_/°	2.854, 25.388	3.070, 27.641	3.097, 27.602
GOF	1.083	0.996	1.030
*R*_1_, w*R*_2_ [*I* > 2*σ*(*I*)]^a^	0.0694, 0.1722	0.0883, 0.1995	0.0592, 0.1554

a *R*_1_ = ∑||*F*_0_| − |*F*_c_||/∑|*F*_0_| and w*R*_2_ = {∑[w(*F*_0_^2^ − *F*_c_^2^)^2^]/∑[w(*F*_0_^2^)^2^]}^1/2^.

## Results and discussion

### Preparation of CPs

CP 1, [Mg(H_2_EBTC)(DMF)_2_(H_2_O)_2_], was obtained under the same reaction conditions as those used for the preparation of a Mg-based CP, [Mg(H_2_EBTC)(DMF)_2_], which we previously reported^25^ except that 2-aminoterephthalic acid (2-APA) was added during the synthesis of 1. In addition, CP 2 and 3 were also generated under the same solvothermal conditions but with an elevated temperature of 110 °C, and the difference between the preparation processes of 2 and 3 is that only 4-aminobenzenesulphonic acid (4-ABA) was added during the synthesis of 2. Notably, it is not possible to achieve 1 (or 2) but instead only [Mg(H_2_EBTC)(DMF)_2_]^25^ (or 3) is formed in the absence of 2-APA (or 4-ABA) during the reaction process. These findings indicated that 2-APA and 4-ABA induced the formation of the crystal structures of 1 and 2 in the corresponding solvothermal reactions and acted as SIAs in the crystal growth process.

### Crystal structures

**Fig. 1:**
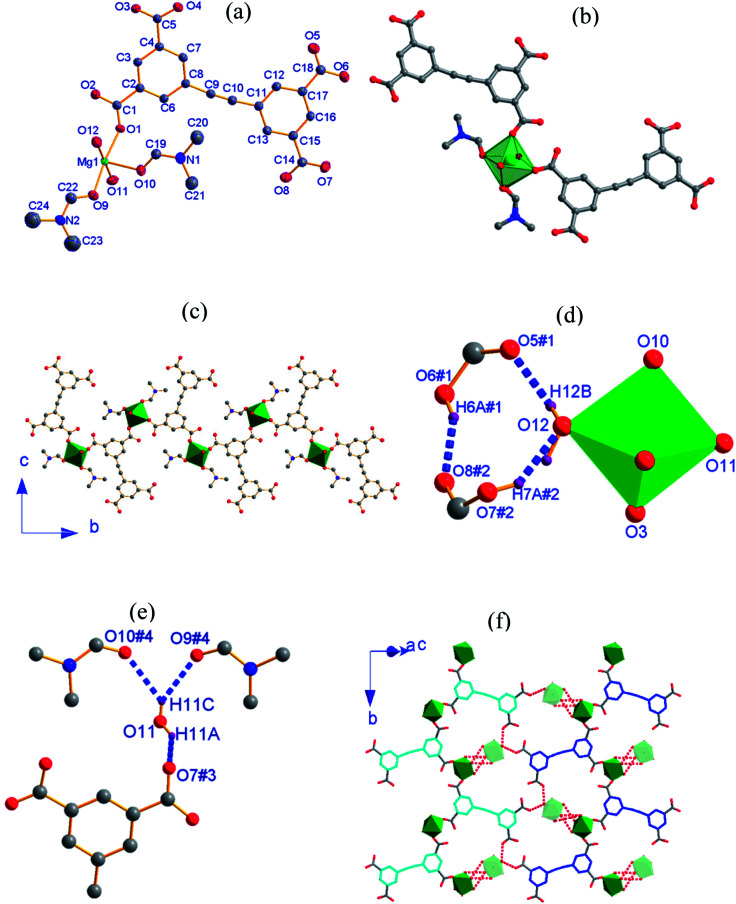
(a) An asymmetric unit of 1 with thermal ellipsoids at 50% probability level where one coordinated DMF molecule is disordered; (b) the coordination environment around the Mg^2+^ ion; (c) 1-D chain structure where the disordered methyl groups in DMF are omitted for clarity; (d) H-bonds between the H_2_O molecule with O12 and COO^−^ groups; (e) H-bonds between the H_2_O molecule with O11 and COO^−^ groups as well as the O atoms of DMF molecules; (f) molecular layers formed *via* H-bonding interactions (the symmetric codes in (d and e): #1 = 1 − *x*, 1 − *y*, 1 − *z*; #2 = 1.5 + *x*, 0.5 − *y*, −0.5 + *z*; #3 = −0.5 + *x*, 0.5 − *y*, −0.5 + *z*; #4 = 1 − *x*, −*y*, 1 − z).

CP 1, [Mg(H_2_EBTC)(DMF)_2_(H_2_O)_2_], crystallizes in the monoclinic space group *P*2_1_/*n* with the formula of C_24_H_26_MgN_2_O_12_. As shown in Fig. 1a, its asymmetric unit contains one Mg^2+^ ion, one H_2_EBTC^2−^ ligand together with two differently coordinated water molecules and two crystallographically inequivalent coordinated DMF molecules. One of the two distinct DMF molecules containing O9 is disordered with two possible positions, and the occupied factors are refined for each possible position. The Mg^2+^ ion is six-coordinated by oxygen atoms, in which two oxygen atoms are obtained from two H_2_EBTC^2−^ ligands (where two COO^−^ groups adopt the *η*^1^ binding mode), two are obtained from two terminal DMF molecules and the other two from two H_2_O molecules, presenting a distorted octahedral geometry (Fig. 1b). The oxygen atoms from the two crystallographically different DMF molecules and the two oxygen atoms from the two crystallographically equivalent H_2_EBTC^2−^ ligands lie in the equatorial plane of the coordination octahedron of MgO_6_. Two DMF molecules and two H_2_EBTC^2−^ ligands adopt a *cis* arrangement, whereas the two crystallographically different water molecules are located in two axial positions of the coordination octahedron of MgO_6_ in a *trans* fashion (ref. Fig. 1b). The Mg–O bond lengths range from 2.055(4) to 2.092(3) Å, and the O–Mg–O bond angles range from 85.72(15) to 99.16(14)° with two coordinated oxygen atoms in a *cis* arrangement; the bond angles also range from 172.88(15) to 179.21(17)° with two coordinated oxygen atoms in a *trans* arrangement in the MgO_6_ coordination octahedron. In the previously reported crystal structure of [Mg(H_2_EBTC)(DMF)_2_],^25^ two coordinated DMF molecules also remain in the *cis* coordination sites in the MgO_6_ coordination octahedron; this is similar to that in 1. Notably, the O–Mg–O bond angles span from 87.05(6) to 98.23(6)° with two coordinated oxygen atoms in a *cis* arrangement and from 172.36(6) to 172.42(8)° with two coordinated oxygen atoms in a *trans* arrangement in the MgO_6_ coordination octahedron, showing more narrow distribution than those in 1; the Mg–O bond distances vary from 2.070(18) to 2.102(20) Å and are slightly longer than those in 1. The steric hindrance between the four *η*^1^-binding mode COO^−^ groups around the Mg^2+^ center probably corresponds to the fact that [Mg(H_2_EBTC)(DMF)_2_], as previously reported, has longer Mg–O bond distances than 1 (where there are two COO^−^ groups with *η*^1^ binding mode around the Mg^2+^ center).

Two phenyl rings in the H_2_EBTC^2−^ ligand are almost coplanar in 1, with a small dihedral angle of 1.3° (ref. Fig. S2a†). The H_2_EBTC^2−^ anion serves as a μ_2_−η^1^:η^1^ mode bridged ligand, and its two COO^−^ groups in the same phenyl ring link two Mg^2+^ ions in the monodentate mode to generate a 1-D zigzag chain along the *b*-axis (Fig. 1c). The H-bonding interactions are observed between the coordinated H_2_O molecules and the coordinated DMF molecules as well as between the coordinated H_2_O molecules and COO^−^ groups; these are displayed in Fig. 1d and e, and the H-bond parameters are listed in Table 2. As depicted in Fig. 1f, the neighboring 1-D zigzag MgO_6_ coordination octahedral chains, where the corresponding phenyl rings are presented in blue and cyan colors, extend into the 2-D supramolecular layer *via* intermolecular H-bonds, and this type of a 2-D supramolecular layer is parallel to the crystallographic (1 0 −1) plane. The adjacent supramolecular layers are further connected to the 3-D framework through H-bonds between the coordinated H_2_O molecules with O11 in MgO_6_ (green color) and the O atoms in DMF molecules with O9 and O10 of the neighboring MgO_6_ (light green color) along the <1 0 −1> direction, which is shown in Fig. 1f.

**Table 2:** Parameters of H-bonds in 1^a^

D–H⋯A	D–H/Å	H⋯A/Å	D⋯A/Å	∠D–H⋯A/°
O12–H12B⋯O5#1	0.85	2.25	2.708(4)	114
O6#1–H6A#1⋯O8#2	0.82	1.75	2.475(4)	146
O7#2–H7A#2⋯O12	0.85	1.94	2.648(4)	144
O11–H11A⋯O7#3	0.95	1.92	2.768(4)	148
O11–H11C⋯O9#4	0.79	2.41	3.095(4)	146
O11–H11C⋯O10#4	0.79	2.44	3.084(4)	139

a Symmetry codes: #1 = 1 − *x*, 1 − *y*, 1 − *z*; #2 = 1.5 + *x*, 0.5 − *y*, −0.5 + *z*; #3 = −0.5 + *x*, 0.5 − *y*, −0.5 + *z*; #4 = 1 − *x*, −*y*, 1 − *z*.

**Fig. 2:**
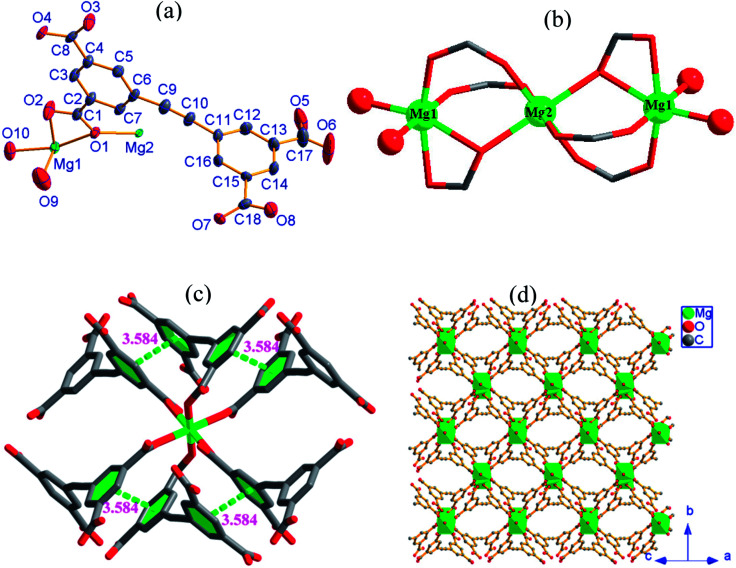
(a) An asymmetric unit with thermal ellipsoids drawn at 50% probability level; (b) trinuclear cluster as SBU with magnesium as assigned in the ball model; (c) intramolecular π⋯π interactions; (d) 2-D network viewed along the <1 0 1> direction (all H atoms were omitted for clarity) in 2.

2, [Mg_3_(HEBTC)_2_(H_2_O)_4_]·(DMF)_3_(H_2_O)_5_, crystallizes in the monoclinic space group *P*2_1_/*n* with the formula C_36_H_22_Mg_3_O_20_. As shown in Fig. 2a, its asymmetric unit consists of two crystallographically independent Mg^2+^ ions (labeled as Mg1 and Mg2) and one partly deprotonated HEBTC^3−^ ligand together with two coordinated water molecules as well as heavy disordered lattice solvents. Mg1 is located at a general position, whereas Mg2 occupies an inversion center. Both Mg1 and Mg2 are coordinated with six oxygen atoms; however, they show significantly different coordination environments. The Mg1 coordination sphere can be viewed as a distorted octahedron, where four coordinated oxygen atoms are obtained from three carboxylates in three HEBTC^3−^ ligands, and the other two coordinated oxygen atoms are provided by two dangling coordinated water molecules. As depicted in Fig. 2b, two coordinated water molecules adopt a *cis* arrangement in the MgO_6_ coordination octahedron with Mg1. The Mg2 coordination sphere displays regular octahedral geometry with *C*_i_ point group symmetry, and six coordinated oxygen atoms are obtained from six carboxylates in six different HEBTC^3−^ ligands (Fig. S3b†). The Mg–O distances range from 1.965(3) to 2.211(3) Å in the coordination octahedron with Mg1 and from 2.028(3) to 2.144(2) Å in the coordination octahedron with Mg2; the O–Mg–O bond angles range from 60.03(10) to 102.05(1)°/161.17(13) to 177.45(16)° with two O atoms in *cis*/*trans* arrangements in MgO_6_ containing Mg1 *versus* 87.07(10)–92.93(10)°/180° with two O atoms in *cis*/*trans* arrangements in MgO_6_ containing Mg2. These parameters in the MgO_6_ coordination octahedra are comparable to the values of the Mg^2+^-carboxylate coordination compounds previously reported.^25^

In the crystal structure of 2, as shown in Fig. 2b, each Mg2 ion is bridged to two neighboring Mg1 ions by four –O–C–O– bridges and two μ_2_-O_carboxyl_ atoms to afford a centrosymmetric trinuclear {Mg_3_} cluster as secondary building unit (SBU), where three Mg^2+^ ions are strictly linear because Mg2 is located at an inversion center; the Mg1⋯Mg2 distance is 3.602 Å. As shown in Fig. S2b,† two phenyl rings in HEBTC^3−^ exhibit a dihedral angle of 82.3°; this indicates a strong spatial-distortion effect from coordination with Mg^2+^ ions, presumably to accommodate the steric demands of the dense SBUs. Two of the three COO^−^ groups adopt (μ_2_−η^1^, η^1^) binding modes to connect one Mg1 and one Mg2, and the remaining COO^−^ group in the HEBTC^3−^ ligand shows (μ_2_−η^2^, η^1^) binding mode to coordinate to one Mg1 and one Mg2. Each HEBTC^3−^ ligand links six Mg^2+^ ions through (μ_2_−η^1^, η^1^) and (μ_2_−η^2^, η^1^) coordination modes, and it also serves as a μ_6_-bridge linker. As shown in Fig. 2c, the neighboring phenyl rings from different HEBTC^3−^ ligands show face-to-face alignment with a centroid-to-centroid distance of 3.584 Å, indicating the existence of π⋯π interactions between them. The linear {Mg_3_} cluster links six neighbors by six HEBTC^3−^ ligands, and each HEBTC^3−^ ligand connects three adjacent trinuclear SUBs to form a 2-D metal–organic framework, which is parallel to the crystallographic *bc*-plane, as depicted in Fig. 2d. The coordinated H_2_O molecules and the COOH groups in HEBTC^3−^ ligands distribute on the surfaces of the 2-D frameworks. The heavy disordered lattice solvent molecules are residual in the pores of the frameworks.

**Fig. 3:**
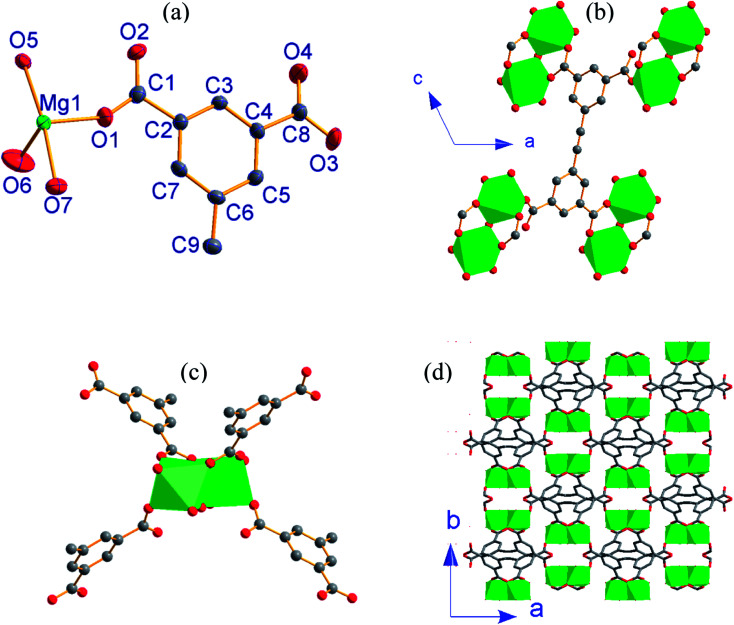
(a) An asymmetric unit of 3 with thermal ellipsoids drawn at 50% probability level; (b) connectivity of EBTC^4−^ ligand; (c) connectivity of binuclear cluster as SBU; (d) 3-D packing framework viewed along the *c*-axis (all H atoms are omitted for clarity) in 3.

3, [Mg_2_(EBTC)(H_2_O)_5_]·(DMF)(H_2_O), crystallizes in the monoclinic space group *C*2/*c* with the formula C_18_H_16_Mg_2_O_13_. As shown in Fig. 3a, its asymmetric unit consists of one Mg^2+^ ion and half H_2_EBTC^2−^ ligand together with three coordinated water molecules as well as the heavy disordered lattice solvents. The Mg^2+^ ion is six-coordinated and connected by three oxygen atoms from three different H_2_EBTC^2−^ ligands and three oxygen atoms from three H_2_O molecules. Two neighboring MgO_6_ coordination octahedra share a vertex (O5) to form a binuclear [Mg_2_O(COO)_2_] SBU with Mg1⋯Mg1 distance of 3.556 Å (Fig. 3b).

**Fig. 4:**
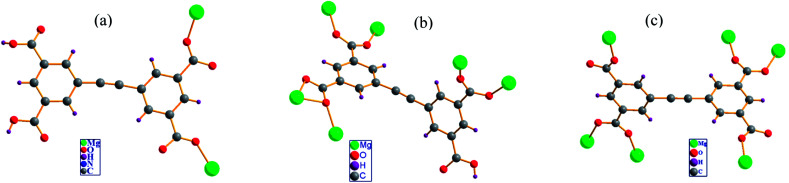
Coordination modes of the H_2_EBTC^2−^/HEBTC^3−^/EBTC^4−^ ligand observed in the crystal structures of (a) 1 (b) 2 and (c) 3.

The EBTC^4−^ ligand shows *C*_2_ point group symmetry and a two-fold rotation axis parallel to the *b*-axis, which passes through the midpoint of the C≡C triple bond. The dihedral angle of the two phenyl rings in EBTC^4−^ is 83.8°, indicating a strong spatial-distortion effect from coordination with the Mg^2+^ ions to accommodate the steric demands of the dense SBUs (ref. Fig. S2c†). Four carboxylate groups in the EBTC^4−^ ligand adopt two different coordination modes μ_1_−η^1^:η^0^ and μ_2_−η^1^:η^1^ (Fig. 4c). The EBTC^4−^ ligand connects four binuclear [Mg_2_O(COO)_2_] SBUs, and the binuclear [Mg_2_O(COO)_2_] SBU connects four EBTC^4−^ ligands (ref. Fig. 3c) to extend into a 3-D framework, which is displayed in Fig. 3d projected along the *c*-axis and Fig. S4b and S4c† viewed along the *b*-axis and the <1 0 1*>* direction, respectively. The heavy disordered lattice solvent molecules are located in the pores of the 3-D framework.

### Diversity of coordination mode of H_4_EBTC

In the crystal structures of 1–3, H_2_EBTC^2−^/HEBTC^3−^/EBTC^4−^ ligands formed as a result of deprotonation of the H_4_EBTC ligand during solvothermal synthesis show diverse coordination modes to that of the Mg^2+^ ion. As shown in Fig. 4, the COOH group is uncoordinated to Mg^2+^ ion (in both 1 and 2), and the deprotonated COO^−^ groups show three types of coordination modes, *i.e.*, monodentate μ_1_−η^1^:η^0^ in 1 and 3 and bidentate μ_2_−η^1^:η^1^ in 2 and 3 as well as μ_2_−η^2^:η^1^ in 2. H_2_EBTC^2−^ acts as a μ_2_-bridging ligand in 1; HEBTC^3−^ serves as a μ_6_-bridging ligand in 2, and EBTC^4−^ also acts as a μ_6_-bridging ligand in 3. Thus, the different deprotonation degrees of the carboxylic groups can facilitate the modification of coordination types, leading to the assembly of metal–organic nets with different structures. In addition, the dihedral angles between the two phenyl rings in the H_2_EBTC^2−^/HEBTC^3−^/EBTC^4−^ ligand vary from 1.3° to 83.8° in 1–3, indicating that strong spatial-distortion effects exist for the H_2_EBTC^2−^/HEBTC^3−^/EBTC^4−^ ligand, which allow compliance with coordination with Mg^2+^ ions to accommodate the steric demands of the dense SBUs. This fact demonstrates that the rotation of the two phenyl rings around the –C≡C– group only needs to overcome a small energy barrier, and such a feature makes the H_2_EBTC^2−^/HEBTC^3−^/EBTC^4−^ ligand rather flexible to fit to different coordination modes with the metal centers in the solvothermal process. Therefore, the flexible molecular conformation and various coordination modes of the H_2_EBTC^2−^/HEBTC^3−^/EBTC^4−^ ligand with metal centers result in the structural diversity of 1–3.

### Possible role of 2-APA or 4-ABA in the crystal growth

By comparison of the crystal structures of 1 and [Mg(H_2_EBTC)(DMF)_2_]^25^ as well as 2 and 3, it is found that SIA 2-APA or 4-ABA leads to reduced dimensionality of the crystal structure of the final solvothermal reaction product; *e.g.*, [Mg(H_2_EBTC)(DMF)_2_]^25^ and 3 show structures with 3-D metal–organic frameworks (without SIA), whereas 1 and 2 display structures with a 1-D coordination polymer chain (with 2-APA at 85 °C) and 2-D coordination layer (with 4-ABA at 110 °C), respectively, which presents a good case of SIA effects. At the present stage, it remains unclear what exactly is the critical role played by 2-APA or 4-ABA in the formation process of the crystal structures of 1 or 2. It is well-known that the formation of single crystals includes two steps of important processes, namely, initial formation and growth of nuclear crystals. It is impressive that zwitterionic surfactants are widely used for tuning the morphology of nanocrystals during the process of crystal growth in the nanomaterial area, and the surfactants prefer to selectively adsorb on some certain crystallographic planes, which prevents further growth of the adsorbed crystallographic planes to give the special morphology of the nanocrystals. The zwitterionic 2-APA or 4-ABA induces the generation of a new crystal structure under almost the same solvothermal reaction conditions, demonstrating that the 2-APA or 4-ABA molecules certainly participate in the initial formation process of nuclear crystals. Notably, with respect to the crystal structure dimensionalities of [Mg(H_2_EBTC)(DMF)_2_]^25^ and 3 obtained in the absence of 2-APA or 4-ABA, the crystal structure dimensionalities of 1 and 2, which are achieved in the presence of 2-APA or 4-ABA, are reduced, and this finding reveals that the H-bonding interactions between the carboxylates in the H_2_EBTC^2−^ ligand and the protonated amino groups in 2-APA or 4-ABA probably play a specific role in the crystal nucleation process. Such types of H-bonding interactions prevent the carboxylate groups of H_2_EBTC^2−^/HEBTC^3−^ ligand from further coordinating to Mg^2+^ ions to form the crystal structure with higher dimensionality.

### Photoluminescent spectra and photophysical property

**Fig. 5:**
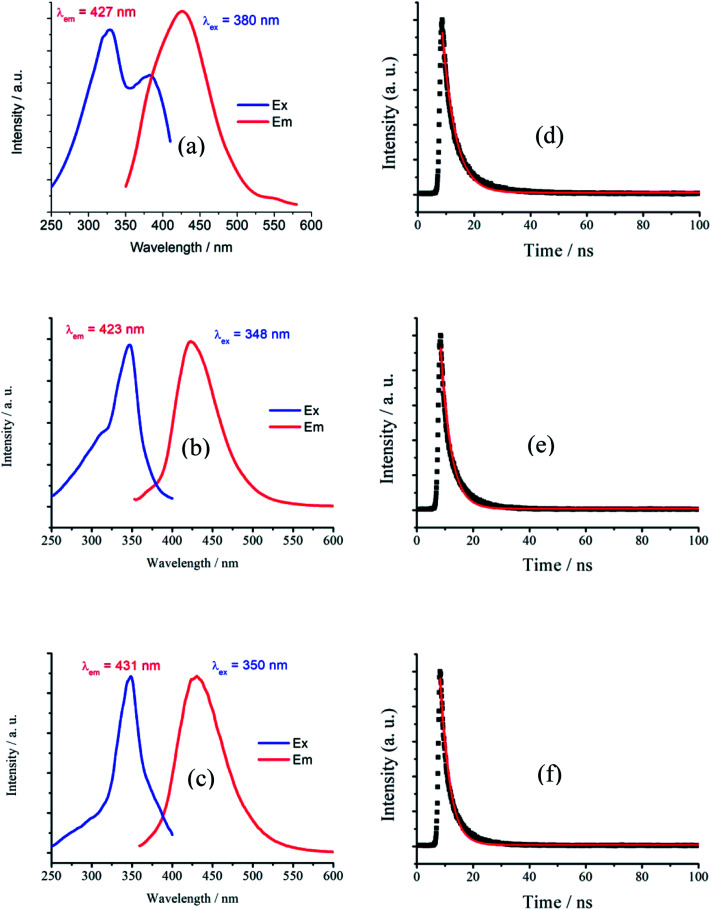
Excitation and emission spectra of (a) 1 (b) 2 (c) 3 and emission decay curves of (d) 1 (e) 2 (f) 3 in the solid state at 298 K.

**Fig. 6:**
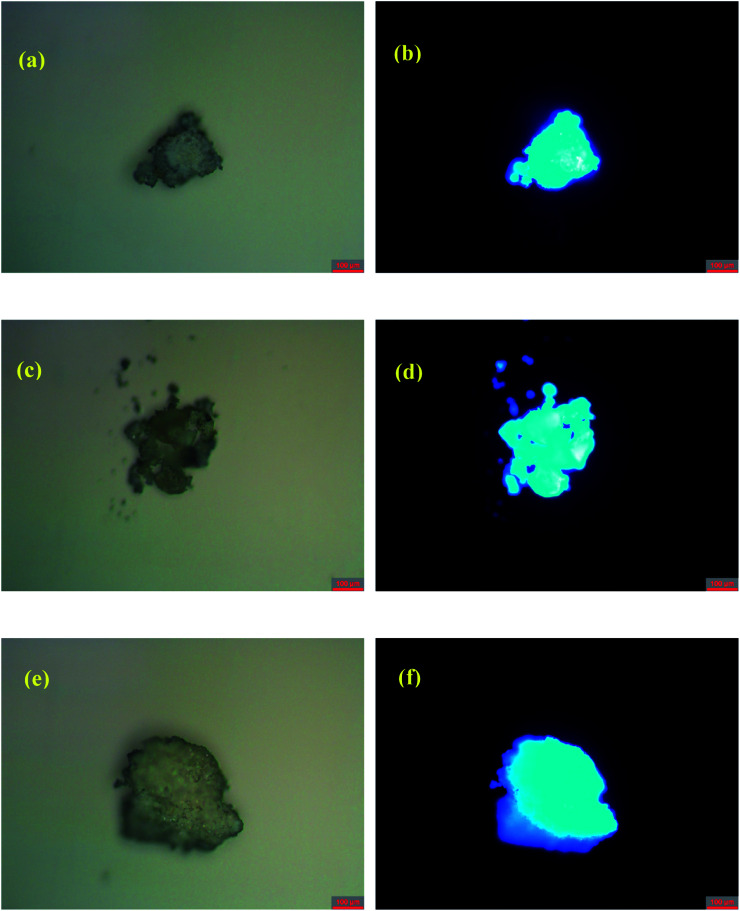
Photos of powders of 1–3 under (a, c and e) ambient light and (b, d and f) ultraviolet irradiation with *λ*_em_ = 340–380 nm, respectively, at room temperature.

The solid-state photoluminescent (PL) properties of 1–3 together with that of the ligand H_4_EBTC were investigated for powdered samples at room temperature including the excitation and emission spectra, photoluminescence decay time and absolute quantum yields. The corresponding spectroscopic and photophysical parameters are summarized in Table 3. In Fig. 5, the emission spectra in the solid state at 298 K show intense luminescence with an emission band centered at 427, 423 and 431 nm for 1–3, and the corresponding Commission Internacionale de I'Eclairage (CIE) coordinates are *ca.* (0.1459, 0.0753), (0.1555, 0.0506) and (0.1538, 0.0657) for 1–3, indicating that 1–3 show blue luminescence. These results are in good agreement with the luminescent images of the polycrystalline powders of 1–3 obtained under ultraviolet light with *λ*_ex_ = 330–380 nm at ambient conditions, as shown in Fig. 6.

**Table 3:** Photophysical parameters for H_4_EBTC and 1–3 at 298 K

Compound	*λ*_ex_/nm	*λ*_em_/nm	*τ*_f_/ns	*ɸ*_f_/%	CIE
1	330	427	4.58	3.6	0.1459, 0.0753
2	347	423	3.66	8.8	0.1555, 0.0506
3	350	431	3.33	7.8	0.1538, 0.0657
H_4_EBTC	278	392	—	0.1	0.1592, 0.0205

It is noted that the centers of the emission bands in 1–3 are close to that in the emission spectrum of H_4_EBTC in solid state, which is due to intraligand π–π* electron transition. By comparison of the observations of the emission spectrum of H_4_EBTC in solid state, the maximum of band redshifts *ca.* 35, 31 and 39 nm in the emission spectra of 1–3, respectively, demonstrating that the formation of Mg–O coordination bonds affects the electronic structure of the H_4_EBTC ligand. These spectral differences may be ascribed to different packing fashions of the organic units between CPs and pristine molecular solid. The emission lifetimes are further investigated, and the corresponding luminescence decay curves are displayed in Fig. 5d–f, respectively. The best fits using a single exponent equation give the emission decay lifetimes of 4.58 ns for 1, 3.66 ns for 2 and 3.33 ns for 3; all these values fall within the time scales of typical fluorescence decay lifetimes of conjugated organic molecules, indicating that the emission in 1–3 corresponds to the electron transition between the S_1_ and S_0_ states of the phenyl rings in the partly/fully deprotonated H_4_EBTC ligand. Besides, the absolute quantum yields are determined by means of the integrating sphere technique and under the excitation light with wavelengths of 330 nm, 347 nm and 350 nm for 1–3, and the absolute quantum yields are 3.6, 8.8 and 7.8%, respectively, at 298 K. As shown in Table 3, the absolute quantum yields of 1–3 are much higher than that of the H_4_EBTC ligand, which primarily stems from the hydrogen bonds/π–π interactions and the increased rigidity of fluorescent linkers as well as the extended π-conjugated system due to the coordination effect of metal ions.^32^ The stronger luminescent intensities of 2 and 3 relative to that of 1 are mainly governed by the differences in the bridging modes of the H_2_EBTC^2−^/HEBTC^3−^/EBTC^4−^ ligand. The H_2_EBTC^2−^ moieties behave as μ_2_-linkers in 1, whereas in 2 and 3, the H_2_EBTC^2−^/EBTC^4−^ spacers feature an intricate μ_6_–coordination fashion, which makes the ligand more rigid, allowing decrease in the vibration-induced deactivation.^33^

## Conclusion

In this study, three coordination polymers of magnesium ion with flexible molecular conformations with the H_2_EBTC^2−^/HEBTC^3−^/EBTC^4−^ ligand were synthesized and structurally characterized. The versatile coordination modes of the carboxylate group with the Mg^2+^ ion as well as the flexible molecular conformation of H_2_EBTC^2−^/HEBTC^3−^/EBTC^4−^ ligand gave rise to diverse crystal structures. It was discovered that the zwitterionic 2-APA (4-ABA) molecules could induce the generation of a new crystal structure in the reaction system under the same solvothermal conditions, and this situation is probably related to the fact that the zwitterionic 2-APA (4-ABA) molecules participate in the initial formation process of nuclear crystals. The current study highlighted the effective tuning of the structures of CPs, which can open new avenues for structure tuning of CPs by structural induction agents. On the other hand, three CPs emitted bright ligand-based luminescence at ambient conditions, and all of them showed much higher absolute quantum yields than H_4_EBTC in solid state at ambient conditions; thus, high performance luminescent materials are probably achieved by the rational design of metal coordination polymers, and they have promising applications in display and sensing technique areas.

## Conflicts of interest

There are no conflicts to declare.

## Supplementary Material

RA-008-C8RA04875D-s001

RA-008-C8RA04875D-s002
