# Supplementary material for: Dimensionality of luminescent coordination polymers of magnesium ions and 1,1′-ethynebenzene-3,3′,5,5′-tetracarboxylic acid modulated by structural inducing agents
Source: RSC Adv. 2018 Jul 16;8(45):25489–99. doi: 10.1039/c8ra04875d (PMC9082528; doi:10.1039/c8ra04875d)
Supplement: RA-008-C8RA04875D-s001 [file RA-008-C8RA04875D-s001.pdf]

Electronic Supplementary Material (ESI) for RSC Advances.
This journal is © The Royal Society of Chemistry 2018

Electronic Supplementary Information (ESI)

Dimensionality of luminescent coordination polymers of magnesium ions and 1, 1'–ethynebenzene–3, 3', 5, 5'–tetracarboxylic acid modulated by structural inducing agents

Zhu−Xi Yang,[a] Yin Qian,[a] Jing−Wei Yu,[a] Lu Zhai,*[a] Wen−Wei Zhang,[b] Xiao−Ming Ren*[a,b,c]

[a]*State Key Laboratory of Materials−Oriented Chemical Engineering and College of Chemistry & Molecular Engineering, Nanjing Tech University, Nanjing 210009, P. R. China*

[b]*State Key Laboratory of Coordination Chemistry, School of Chemistry and Chemical Engineering, Nanjing University, Nanjing 210023 P. R. China*

[c]*College of Materials Science& Engineering, Nanjing Tech University, Nanjing 210009, P. R. China*

Email: zhailu@njtech.edu.cn (LZ); xmren@njtech.edu.cn (XMR)

Contents

Fig. S1 PXRD patterns of (a) **1** (b) **2** (c) **3** as−synthesized samples and simulated one based on the single−crystal structures and (d) TG plots of **1**−**3**, indicating that three CPs start to release the coordination solvents or lattice solvents at room temperature

Fig. S2 Dihedral angle between two phenyl rings in (a) **1** (b) **2** (c) **3**

Fig. S3 The coordination environment of (a) Mg1 and (b) Mg2 (c) trinuclear cluster as node being connected to six $HEBTC^{3-}$ ligands (d) packing diagram viewed along c-axis, which shows the stack of two-dimensional coordination layers, in **2** (all H atoms were omitted for clarity)

Fig. S4 (a) Mg^{2+}-binuclear unit and packing diagrams viewed along (b) b-axis (c) [101] direction in **3**

Fig. S5 IR spectra of **1**–**3** recorded using KBr pellet

Fig. S6 Photographs of single crystals of **1**

Fig. S7 Photographs of single crystals of **2**

Fig. S8 Photographs of single crystals of **3**

Table S1: Selected bond distances (Å) and angles (°) in **1**–**3**

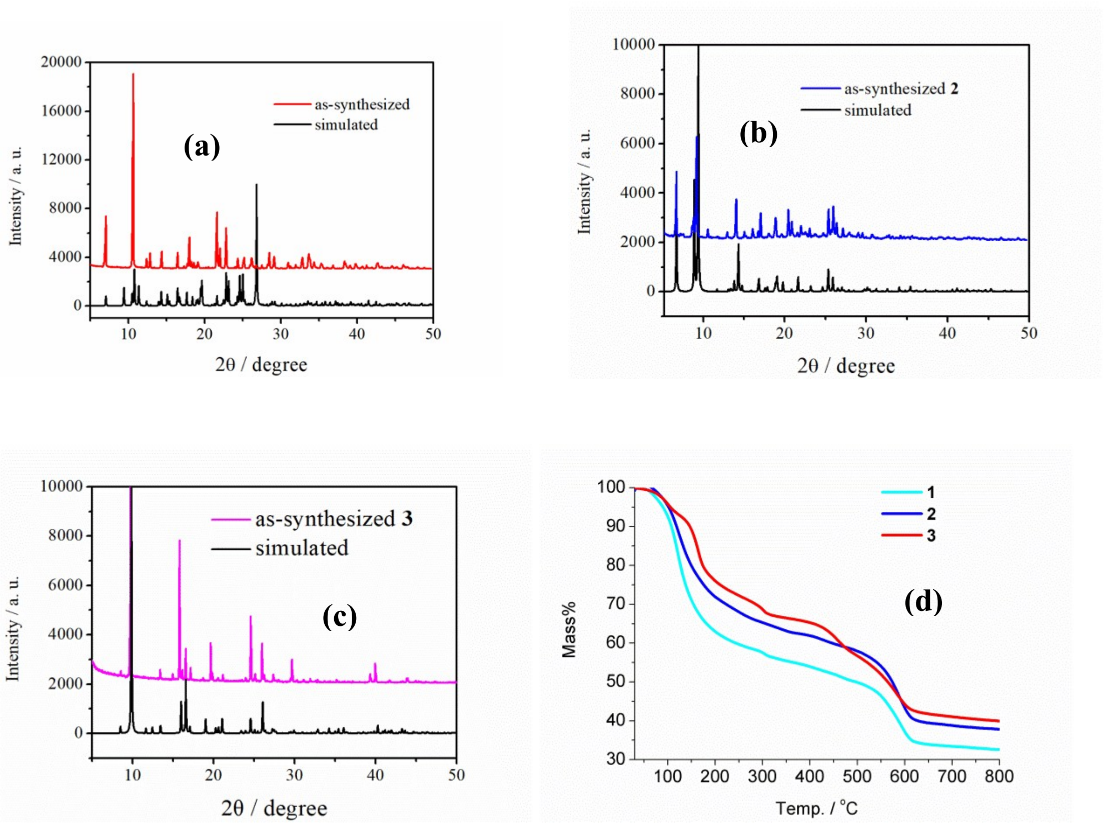

Fig. S1 PXRD patterns of (a) **1** (b) **2** (c) **3** as−synthesized samples and simulated one based on the single−crystal structures and (d) TG plots of **1**−**3**, indicating that three CPs start to release the coordination solvents or lattice solvents at room temperature.

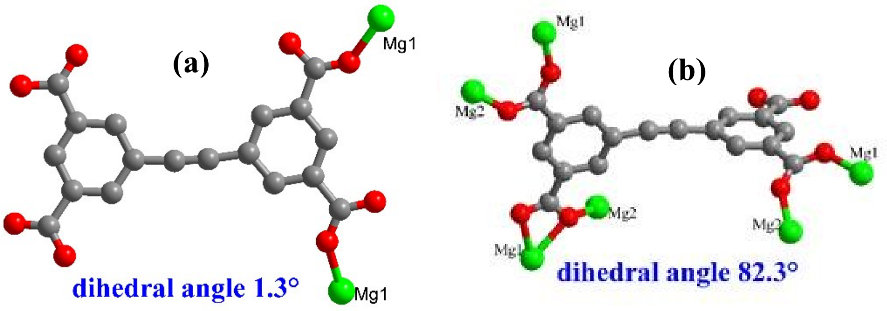

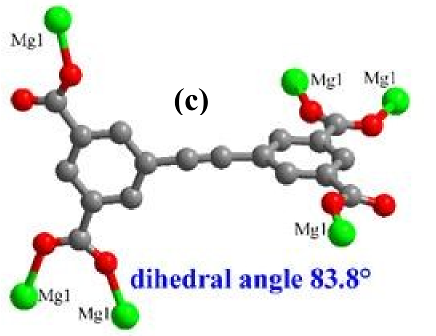

Fig. S2 Dihedral angle between two phenyl rings in (a) **1** (b) **2** (c) **3.**

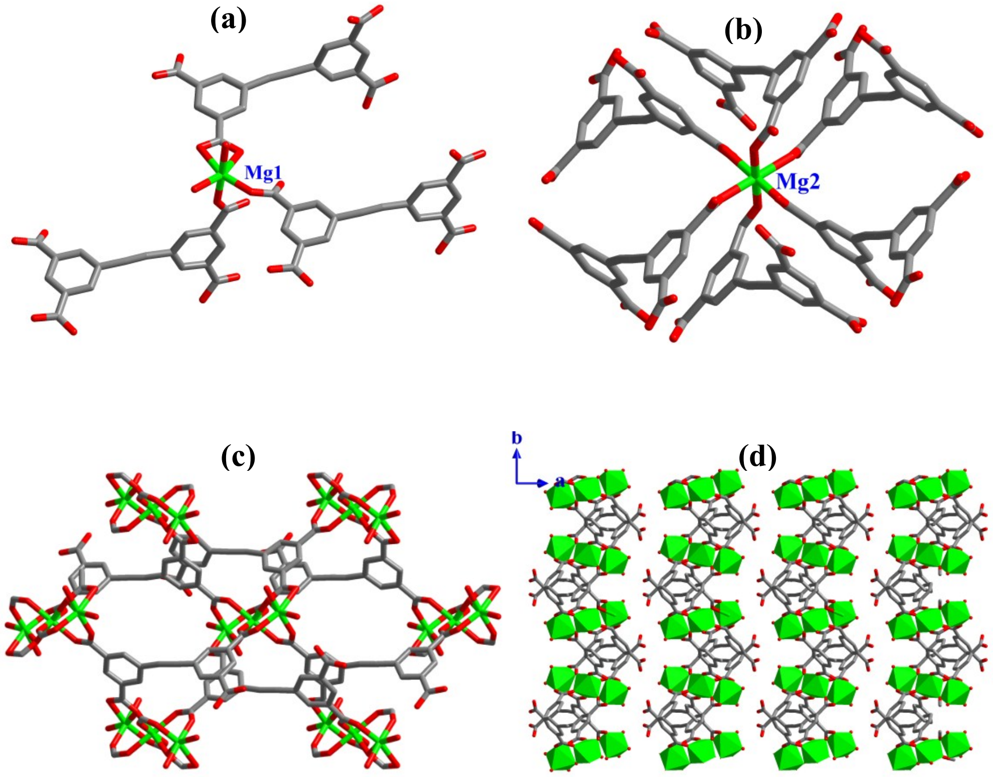

Fig. S3 The coordination environment of (a) Mg1 and (b) Mg2 (c) trinuclear cluster as node being connected to six $HEBTC^{3-}$ ligands (d) packing diagram viewed along c-axis, which shows the stack of two-dimensional coordination layers, in **2** (all H atoms were omitted for clarity).

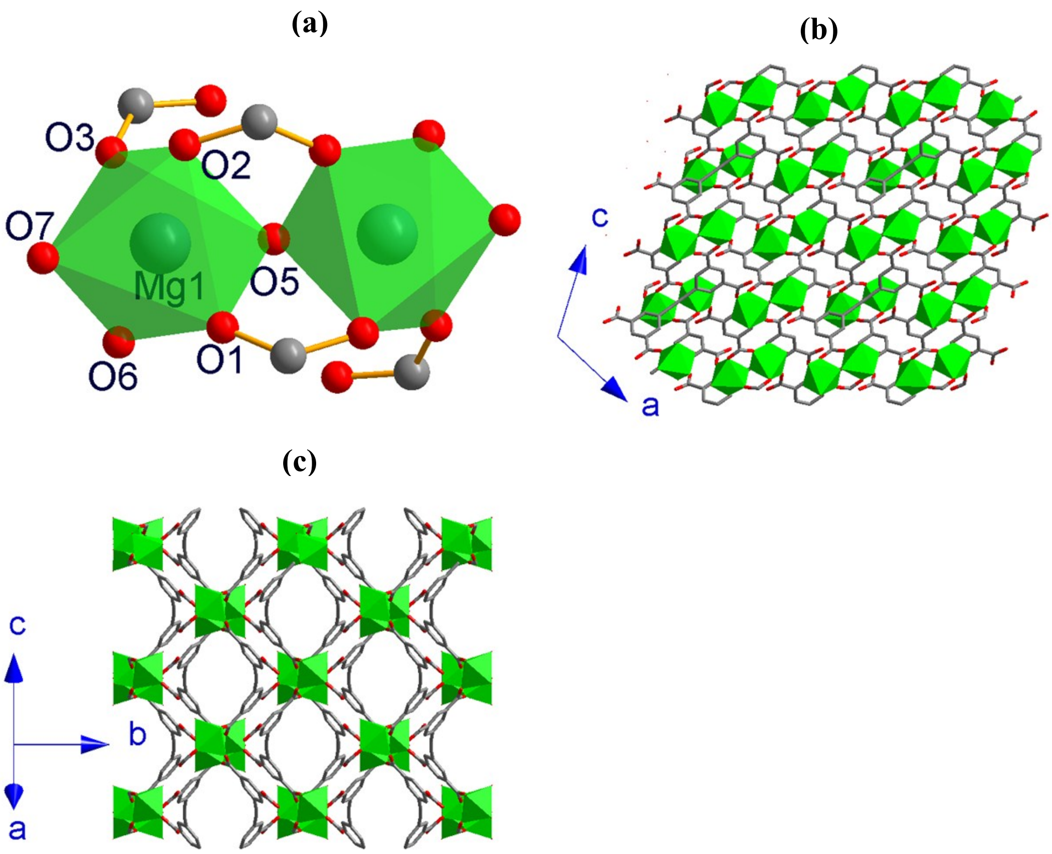

Fig. S4 (a) Mg^{2+}-binuclear unit and packing diagrams viewed along (b) b-axis (c) [101] direction in **3.**

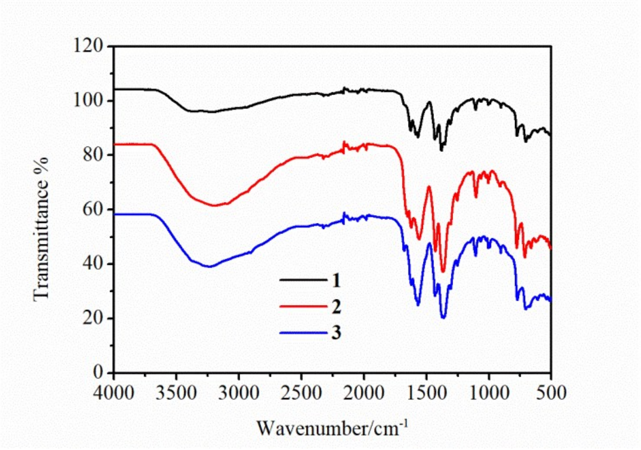

Fig. S5 IR spectra of **1**–**3** recorded using KBr pellet.

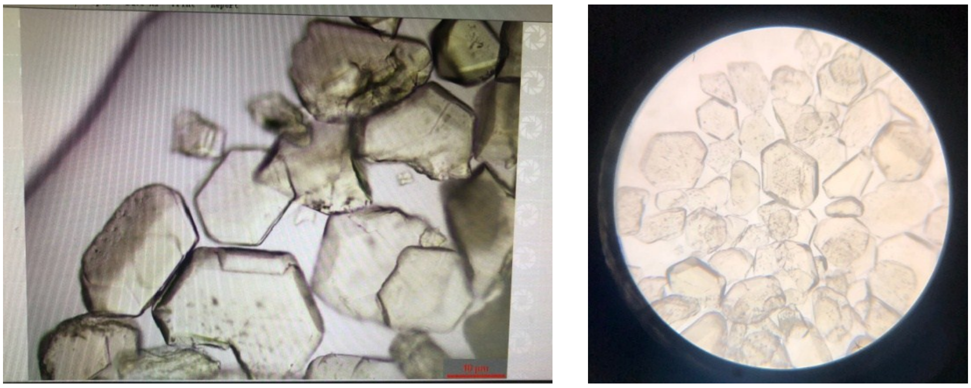

Fig. S6 Photographs of single crystals of **1**.

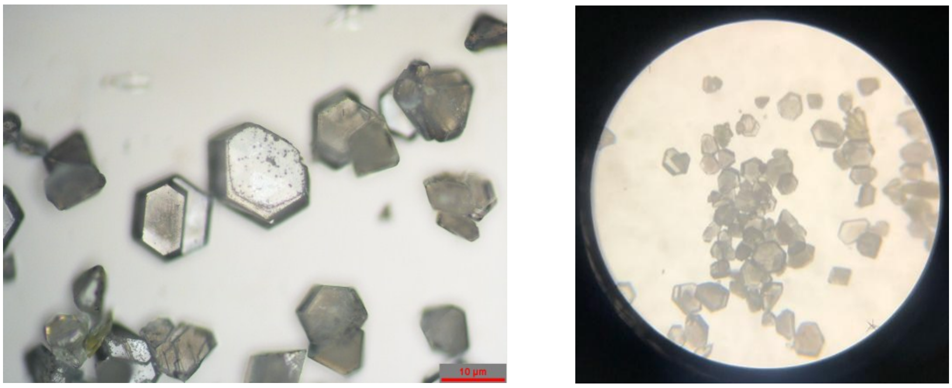

Fig. S7 Photographs of single crystals of **2**.

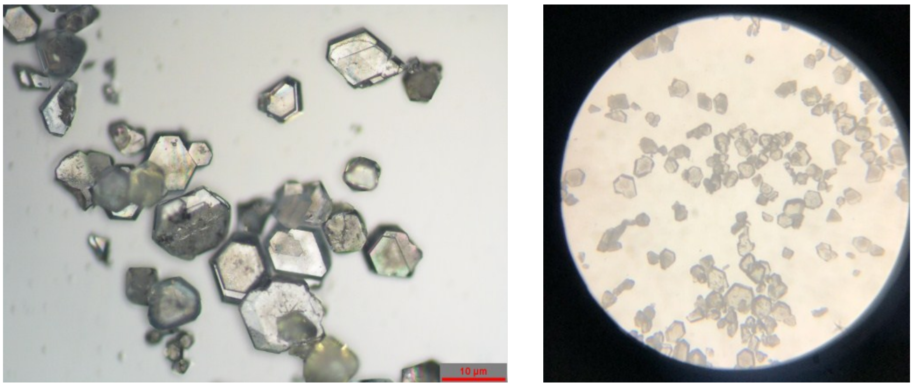

Fig. S8 Photographs of single crystals of **3**.

Table S1: Selected bond distances (Å) and angles (°) in **1–3**

CP 1

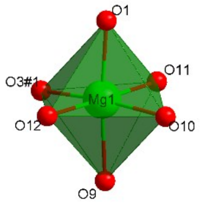

Bond lengths

Mg1–O1	2.006(3)	Mg1–O3#1	2.070(3)	Mg1–O9	2.081(4)
Mg1–O10	2.092(3)	Mg1–O11	2.064(4)	Mg1–O12	2.055(4)

Bond angles

O12–Mg1–O11	179.21(17)	O12–Mg–O1	89.48(14)	O11– Mg1–O1	89.74(14)
O12–Mg1–O3#1	91.47(14)	O11–Mg1–O3#1	88.47(14)	O1–Mg1–O3#1	99.16(14)
O12–Mg1– O9	90.34(14)	O11–Mg1–O9	90.45(15)	O1–Mg1–O9	172.88(15)
O3#1–Mg1–O9	87.97(15)	O12– Mg1–O10	90.45(14)	O11– Mg1–O10	89.69(14)
O1–Mg1–O10	87.16(14)	O3#1– Mg1–O10	173.40(15)	O9– Mg1–O10	85.72(15)

CP 2

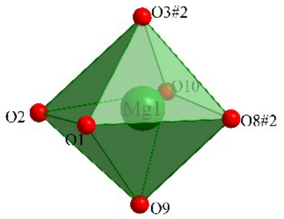

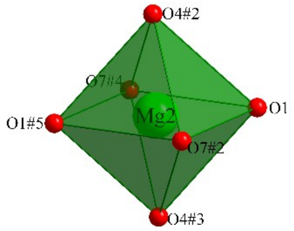

Bond lengths

Mg1–O1	2.153(3)	Mg1–O2	2.211(3)	Mg1–O3#2	1.965(3)

Mg1–O8#2	2.013(3)	Mg1–O9	2.068(4)	Mg1–O10	2.028(3)
Mg2–O1	2.144(2)	Mg2–O1#5	2.144(2)	Mg2–O4#2	2.139(2)
Mg2–O4#3	2.139(2)	Mg2–O7#2	2.028(3)	Mg2–O7#4	2.028(3)

Bond angles

O3#2–Mg1–O8#2	92.63(15)	O3#2–Mg1–O10	90.92(13)	O8#2–Mg1–O10	96.65(14)
O3#2–Mg1–O9	177.45(16)	O8#2–Mg1–O9	89.47(17)	O10–Mg1–O9	87.41(13)
O3#2–Mg1–O1	90.47(12)	O8#2–Mg1–O1	102.05(12)	O10–Mg1–O1	161.17(13)
O9–Mg1–O1	90.47(12)	O3#2–Mg1–O2	102.05(12)	O8#2–Mg1–O2	161.17(13)
O10–Mg1–O2	101.14(12)	O9–Mg1–O2	85.46(16)	O1–Mg1–O2	60.03(10)
O7#4–Mg2–O7#2	180.00(19)	O7#4–Mg2–O4#3	92.93(10)	O7#2–Mg2–O4#3	87.07(10)
O7#4–Mg2–O4#2	87.07(10)	O7#2–Mg2–O4#2	92.93(10)	O4#3–Mg2–O4#2	180.00(18)
O7#4–Mg2–O1#5	87.28(10)	O7#2–Mg2–O1#5	92.72(10)	O4#3–Mg2–O1#5	89.95(9)
O4#2–Mg2–O1#5	90.05(9)	O7#4–Mg2–O1	92.72(10)	O7#2–Mg2–O1	87.28(10)
O4#3–Mg2–O1	90.05(9)	O4#2–Mg2–O1	89.95(9)	O1#5–Mg2–O1	180.0(3)

CP 3

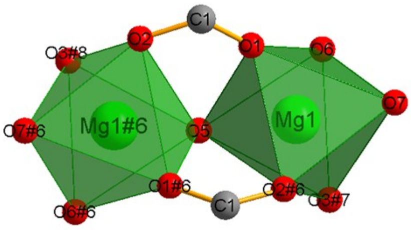

Bond lengths

Mg1–O1	1.9824(19)	Mg1–O2#6	2.1060(19)	Mg1–O3#7	2.0217(19)
Mg1–O5	2.1751(15)	Mg1–O6	2.094(2)	Mg1–O7	2.0819(19)

Mg#6–O1#6	1.9824(19)	Mg#6–O2	2.1060(19)	Mg#6–O3#8	2.0217(19)
Mg#6–O5	2.1751(15)	Mg#6–O6#6	2.094(2)	Mg#6–O7#6	2.0819(19)
		Bond angles			
O1–Mg1–O3#7	172.750(99)	O1–Mg1–O7	95.928(88)	O3#7–Mg1–O7	91.053(87)
O1–Mg1–O6	90.464(104)	O3#7–Mg1–O6	92.018(105)	O7–Mg1–O6	85.651(88)
O1–Mg1–O2#6	91.207(90)	O3#7–Mg1–O2#6	87.261(87)	O7–Mg1–O2#6	86.481(79)
O6–Mg1–O2#6	172.084(100)	O1–Mg1–O5	86.883(64)	O3#7–Mg1–O5	86.501(65)
O7–Mg1–O5	171.129(66)	O6–Mg1–O5	85.915(73)	O2#6–Mg1–O5	101.901(64)
O1#6–Mg1#6–O3#8	172.750(99)	O1#6–Mg1#6–O7#6	95.928(88)	O3#8–Mg1#6–O7#6	91.053(87)
O1#6–Mg1#6–O6#6	90.464(104)	O3#8–Mg1#6–O6#6	92.018(105)	O7#6–Mg1#6–O6#6	85.651(88)
O1#6–Mg1#6–O2	91.207(90)	O3#8–Mg1#6–O2	87.261(87)	O7#6–Mg1#6–O2	86.481(79)
O6#6–Mg1#6–O2	172.084(100)	O1#6–Mg1#6–O5	86.883(64)	O3#8–Mg1#6–O5	86.501(65)
O7#6–Mg1#6–O5	171.129(66)	O6#6–Mg1#6–O5	85.915(73)	O2–Mg1#6–O5	101.901(64)

Symmetry transformations used to generate equivalent atoms for 1/2/3: #1=0.5-x, -0.5+y, 0.5-z; #2=-x, -0.5+y, 0.5-z; #3=x, -0.5-y, -0.5+z; #4=x, 0.5-y, 0.5+z; #5=-x, -y, -z; #6=1-x, y, 1.5-z; #7=-0.5+x, 0.5+y, z; #8=1.5-x, 0.5+y, 1.5-z.
